# Supplementary figures and images for: UBQLN2 restrains the domesticated retrotransposon PEG10 to maintain neuronal health in ALS
Source: eLife. 2023 Mar 23;12:e79452. doi: 10.7554/eLife.79452 (PMC10076021; doi:10.7554/eLife.79452)

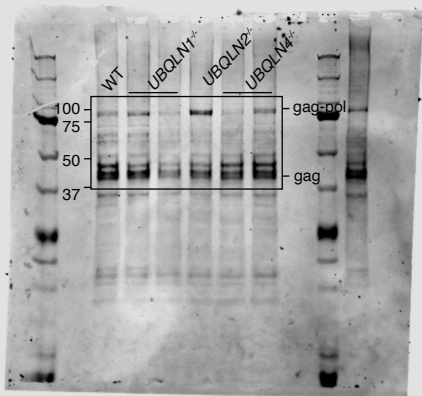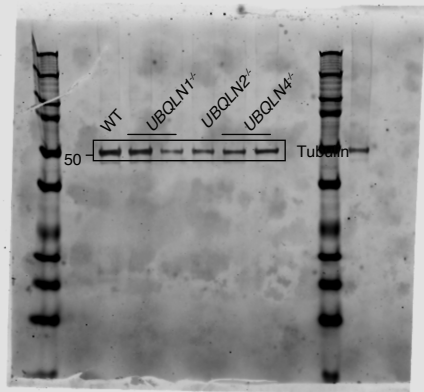

Supplement: Figure 1—source data 1. [file elife-79452-fig1-data1.pdf]

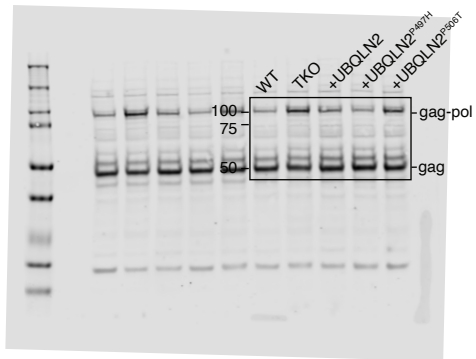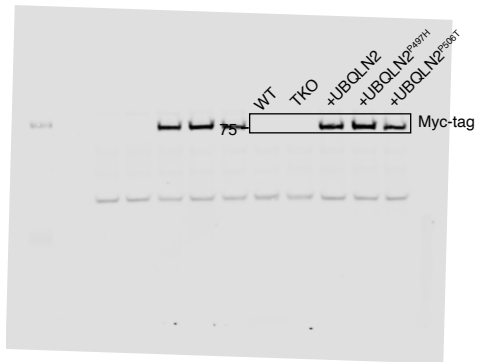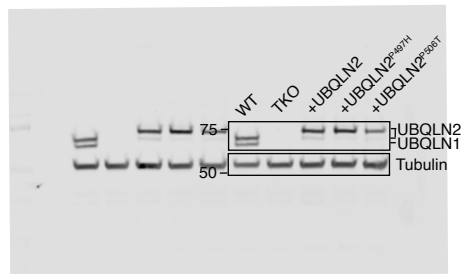

Supplement: Figure 1—source data 2. [file elife-79452-fig1-data2.pdf]

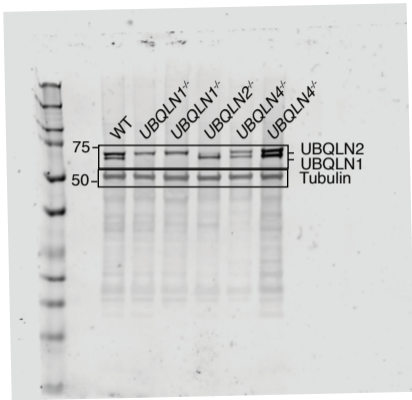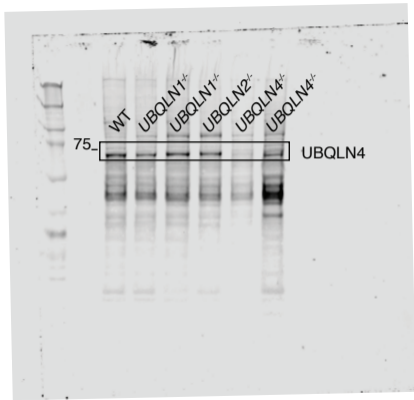

Supplement: Figure 1—figure supplement 1—source data 1. [file elife-79452-fig1-figsupp1-data1.pdf]

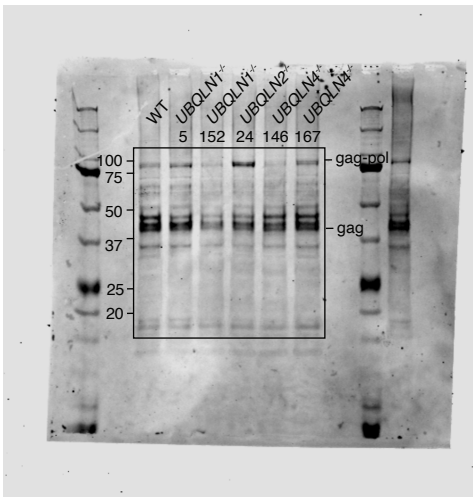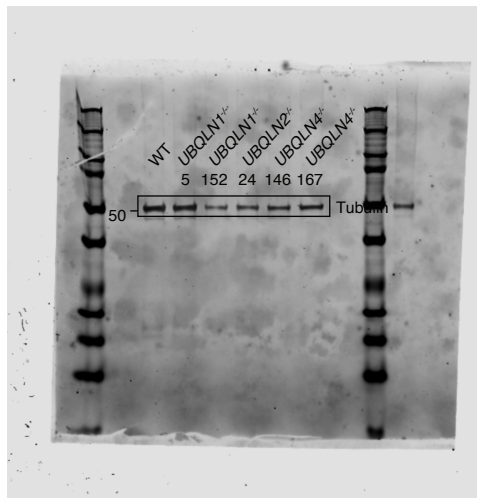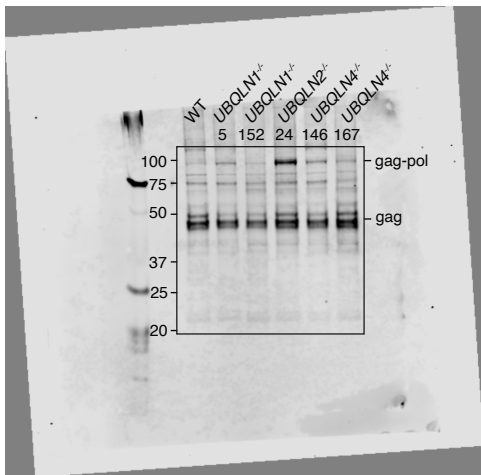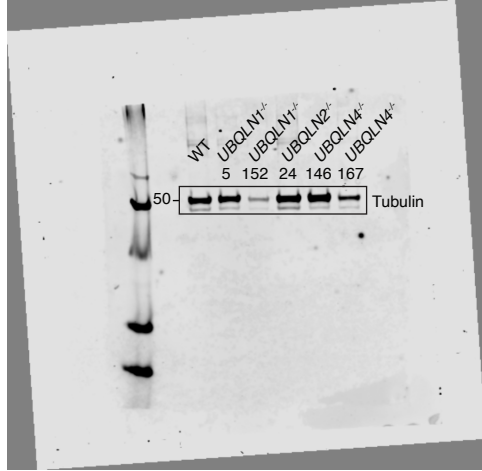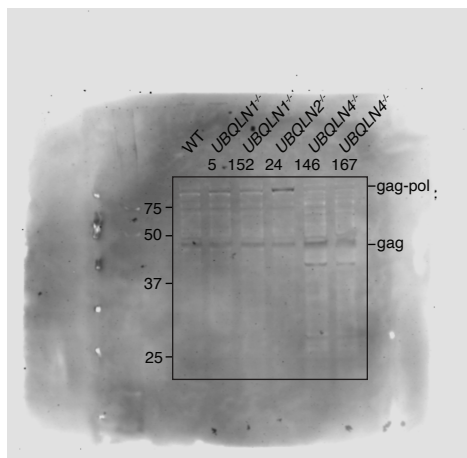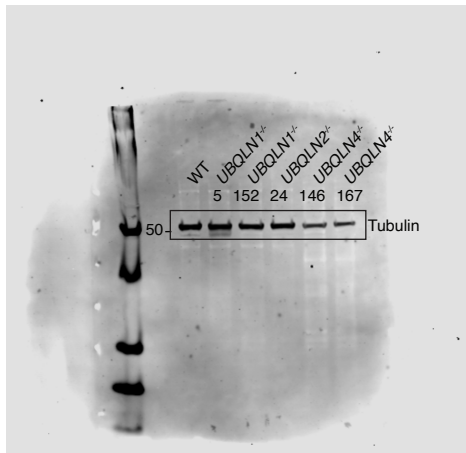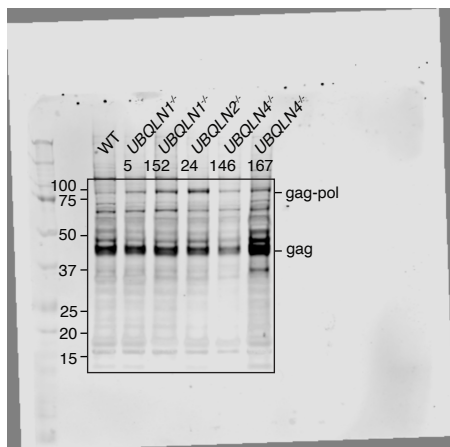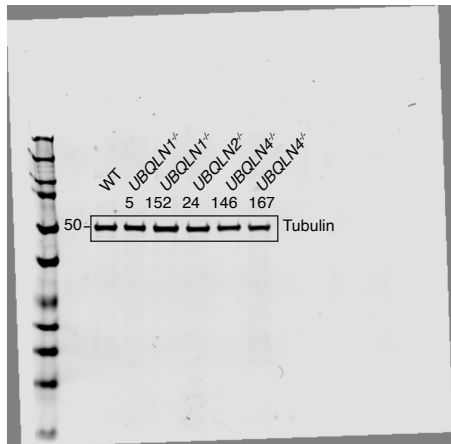

Supplement: Figure 1—figure supplement 1—source data 2. [file elife-79452-fig1-figsupp1-data2.pdf]

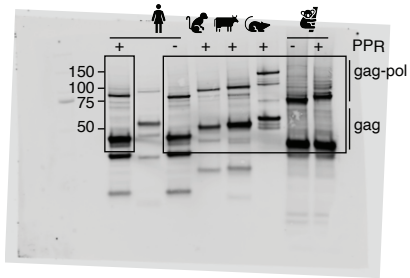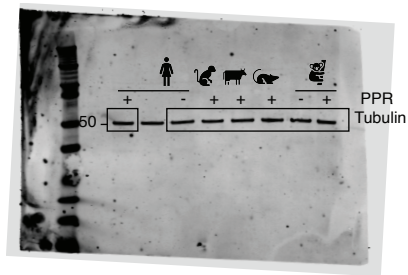

Supplement: Figure 3—source data 1. [file elife-79452-fig3-data1.pdf]

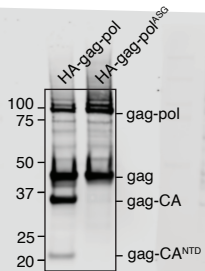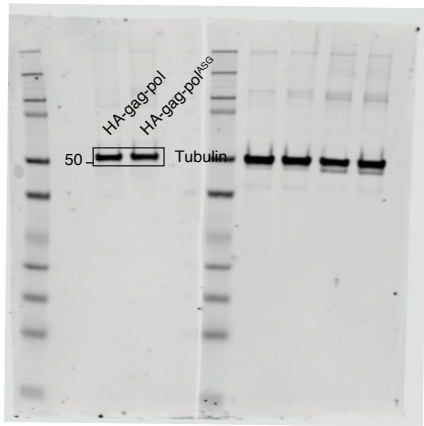

Supplement: Figure 4—source data 1. [file elife-79452-fig4-data1.pdf]

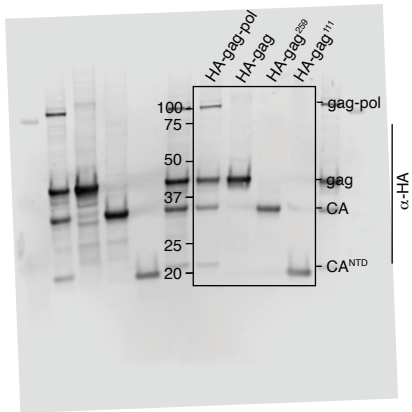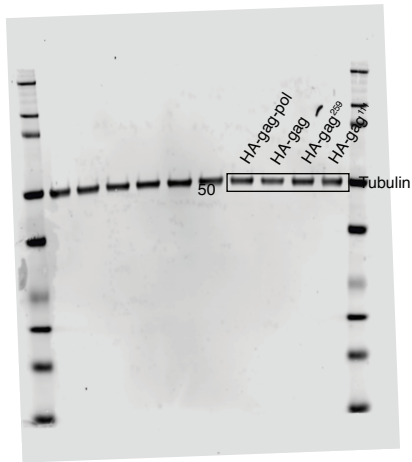

Supplement: Figure 4—source data 2. [file elife-79452-fig4-data2.pdf]

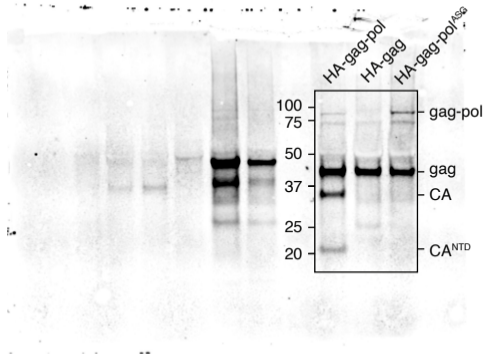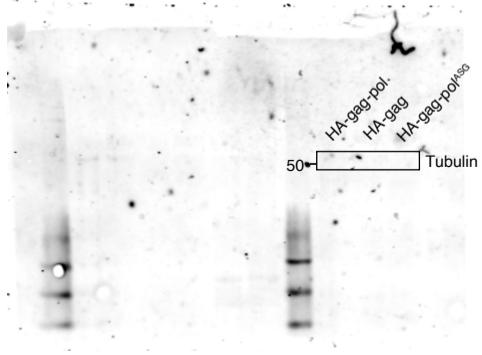

Supplement: Figure 4—source data 3. [file elife-79452-fig4-data3.pdf]

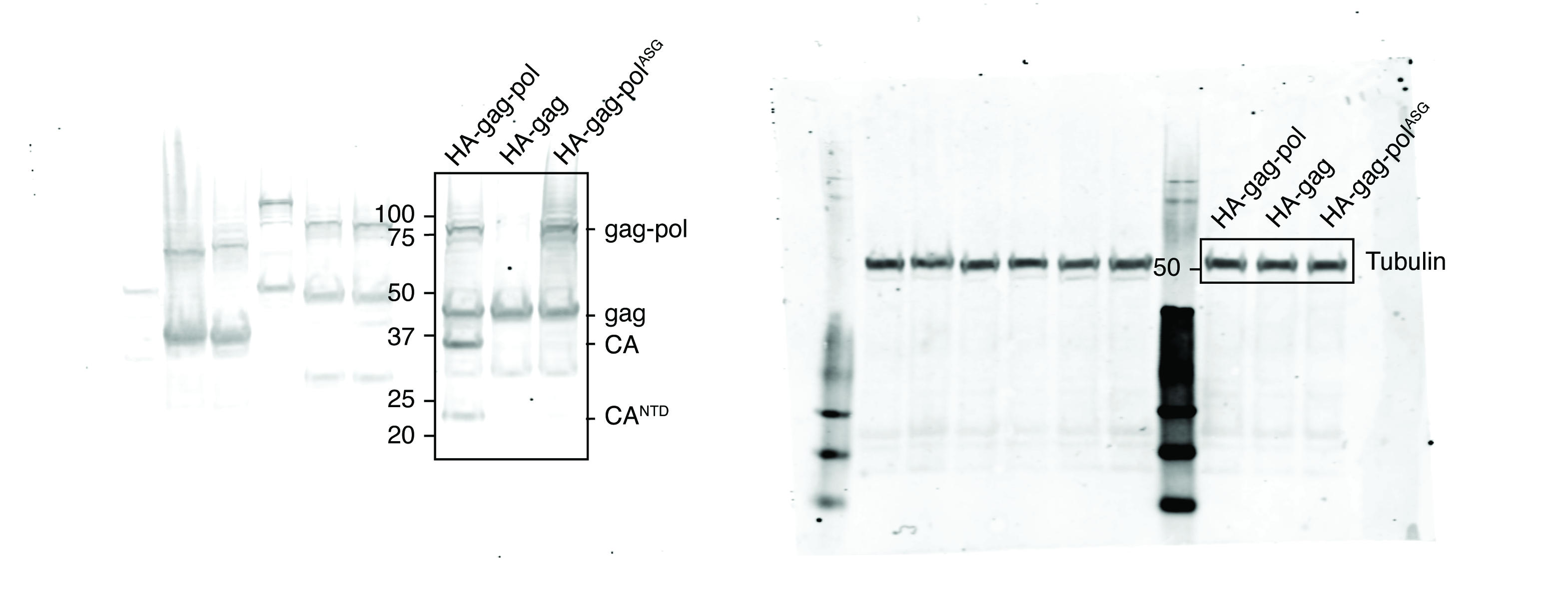

Supplement: Figure 4—source data 4. [file elife-79452-fig4-data4.tif]

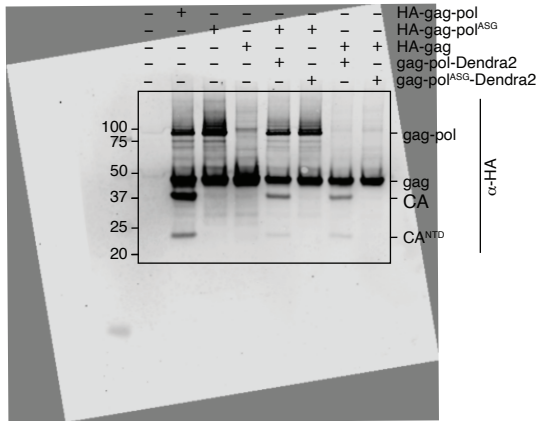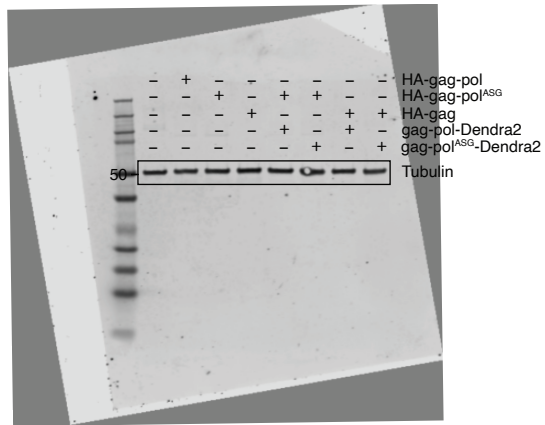

Supplement: Figure 4—source data 5. [file elife-79452-fig4-data5.pdf]

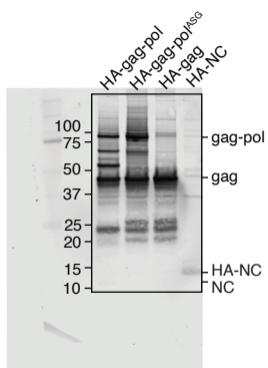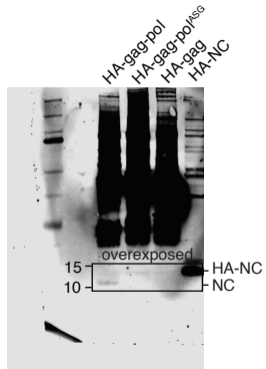

Supplement: Figure 4—source data 6. [file elife-79452-fig4-data6.pdf]

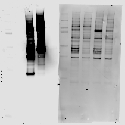

Supplement: Source data 1. [file elife-79452-data1.zip › RawWBimages_eLife resubmission/0000564_02/0000564_02_TH.jpg]

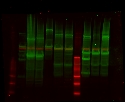

Supplement: Source data 1. [file elife-79452-data1.zip › RawWBimages_eLife resubmission/0000350_02/0000350_02_TH.jpg]

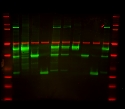

Supplement: Source data 1. [file elife-79452-data1.zip › RawWBimages_eLife resubmission/0000230_02/0000230_02_TH.jpg]

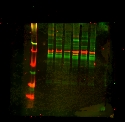

Supplement: Source data 1. [file elife-79452-data1.zip › RawWBimages_eLife resubmission/0000205_02/0000205_02_TH.jpg]

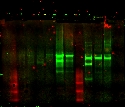

Supplement: Source data 1. [file elife-79452-data1.zip › RawWBimages_eLife resubmission/0000351_02/0000351_02_TH.jpg]

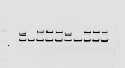

Supplement: Source data 1. [file elife-79452-data1.zip › RawWBimages_eLife resubmission/0000536_01/0000536_01_TH.jpg]

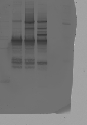

Supplement: Source data 1. [file elife-79452-data1.zip › RawWBimages_eLife resubmission/0000583_01/0000583_01_TH.jpg]

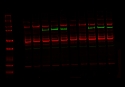

Supplement: Source data 1. [file elife-79452-data1.zip › RawWBimages_eLife resubmission/0000531_01/0000531_01_TH.jpg]

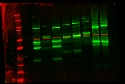

Supplement: Source data 1. [file elife-79452-data1.zip › RawWBimages_eLife resubmission/0000317_02/0000317_02_TH.jpg]

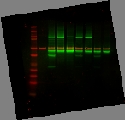

Supplement: Source data 1. [file elife-79452-data1.zip › RawWBimages_eLife resubmission/0000559_03/0000559_03_TH.jpg]

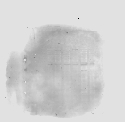

Supplement: Source data 1. [file elife-79452-data1.zip › RawWBimages_eLife resubmission/0000217_02/0000217_02_TH.jpg]

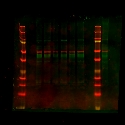

Supplement: Source data 1. [file elife-79452-data1.zip › RawWBimages_eLife resubmission/0000233_02/0000233_02_TH.jpg]

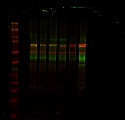

Supplement: Source data 1. [file elife-79452-data1.zip › RawWBimages_eLife resubmission/0000347_02/0000347_02_TH.jpg]

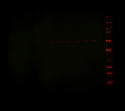

Supplement: Source data 1. [file elife-79452-data1.zip › RawWBimages_eLife resubmission/0000255_01/0000255_01_TH.jpg]
